# Flow dynamics through discontinuous clogs of rigid particles in tapered microchannels

**DOI:** 10.1038/s41598-022-25831-w

**Published:** 2022-12-30

**Authors:** Olukayode T. Majekodunmi, Sara M. Hashmi

**Affiliations:** 1 Department of Chemical Engineering, Northeastern University, Boston, MA 02115 USA; 2 Department of Mechanical and Industrial Engineering, Northeastern University, Boston, MA 02115 USA; 3 Department of Chemistry and Chemical Biology, Northeastern University, Boston, MA 02115 USA

**Keywords:** Chemical engineering, Fluid dynamics, Soft materials, Fluidics

## Abstract

Suspended particles flowing through complex porous spaces exhibit clogging mechanisms determined by factors including their size, deformability, and the geometry of the confinement. This study describes the clogging of rigid particles in a microfluidic device made up of parallel microchannels that taper from the inlet to the outlet, where the constriction width is approximately equal to the particle size. This converging geometry summarizes the dynamics of clogging in flow channels with constrictions that narrow over multiple length scales. Our novel approach allows the investigation of suspension flow dynamics in confined systems where clogs are formed both by sieving and bridging mechanisms simultaneously. Here, flow tests are conducted at constant driving pressures for different particle volume fractions, and a power-law decay which appears to be peculiar to the channels’ tapered geometry is observed in all cases. Compared to non-tapered channels, the power-law behavior shows flowrate decay is significantly weaker in tapered channels. This weaker flowrate decay is explained by the formation of discontinuous clogs within each channel. Micrographs of the clogged channels reveal clogs do not grow continuously from their initial positions around the channels’ outlet. Rather, new clogs spanning the width of the channel at their points of inception are successively formed as the cake grows toward the inlet area in each microchannel. The results show changes in particle volume fraction at constant driving pressure affect the clogging rate without impacting the underlying dynamics. Unexpectedly, analyses of the particles packing behavior in the microchannels, and post-clogging permeability of the microfluidic devices, reveal the presence of two distinct regimes of driving pressure, though only a small portion of the total device volume and channels surface area are occupied by clogs, regardless of the particle volume fraction. This novel investigation of discontinuous clogging over multiple particle diameters provides unique insights into additional mechanisms to control flow losses in filtration and other confined systems.

## Introduction

Clogs are frequently observed in systems and applications involving the transport of colloidal suspensions through porous media or narrow confinements^1^. These include water filters^2,3^, subsurface aquifers and petroleum reservoirs^4^, several manufacturing processes^5,6^, and biophysical systems such as blood circulation^7,8^. Clogging is not entirely an undesirable phenomenon: it has found applications as the working principle of diverse technologies. In healthcare, it has been employed as a biomarker for early screening of disease states and assessment of their severity and treatment methods, based on the deformability of red blood cells^9,10^. Also demonstrated is clogging as an efficient method for the separation of healthy blood cells from diseased ones, including circulating tumor cells^11^. It is important to note that these developments are facilitated by advancements in microfluidic technologies.

Clogs are formed when suspended particles are approximately the same size or larger than the narrowest dimension of a constriction. This form of clogging is known as sieving, and occurs when $\frac{w_c}{d_p}\le 1$, where $w_c$ is the constriction width and $d_p$ is the particle diameter^1^. Clogging can also occur when the number of particles sufficient to form an arch that spans the width of a large constriction arrive at the same instant^12^. This clogging mechanism is known as bridging, and has been observed when $2 \le \frac{w_c}{d_p} \le 5$, with as many as 9 or 10 particles participating in a bridge^13,14^.

In confinements significantly larger than the sizes of the flowing particles ($w_c \gg d_p$), clogs are initiated by adhesive interactions between the particles and the constriction wall^15–17^. The presence of surface interactions may cause continuous particle deposition on the constriction wall, which increasingly narrows the flow path until eventually clogged by a sieving particle or aggregate^18^.

In addition, clogging is useful in applications such as water treatment, food and pharmaceutical processing, and other industrial processes where high-performance porous membranes are used to trap and remove impurities from suspensions. However, high material and energy costs may be incurred in the operation of particle-laden flow processes. This is due to reduced membrane efficiency and lifetime, and the need for frequent cleaning and/or replacement. At constant driving pressures, clogging causes the volumetric flow of a suspension to decline over time as particles lodge or aggregate in the membrane pores^2,19,20^. Such decreases in flowrate necessitates the use of a higher driving pressure to sustain the process, thereby increasing energy costs^2,4^. It may also lead to plant shutdowns in severe cases where flow is completely blocked.

Besides particle size^21^, surface interactions^16^, and deformability^22^, the dynamics and mechanism of clog formation also depend on flow driving forces^23^ and particle shape^24,25^. Much research efforts have been expended towards isolating these parameters, and investigating them singly, to understand their specific contributions to clogging even at single particle or pore scale. These efforts have been reviewed in detail^1^. In one example, reduced asphericity was reported to increase the probability of clogging of rigid particles in both dry granular and particle-laden flows^25^.

The influence of confinement geometry has been shown to be of significant importance^12,18,26–28^. These investigations are made possible by microfluidic models of complex confinements designed to mimic both naturally occurring porous media and engineered systems^29–31^. For instance, the influence of channel shape on clogging rate has been examined in a microfluidic model containing a series of identical constrictions arranged in parallel^27^. This was done by varying the angle of inclination of the constriction entrance between $0^{\circ }$ and $55^{\circ }$. The fraction of the constrictions clogged per unit time decreased as the entrance angle was increased, regardless of the presence of repulsive or attractive interactions between the suspended particles.

Other studies show that differences in confinement geometry can also alter the trend and rate of flowrate decline. An exponential decay of suspension flowrate at constant driving pressures has been reported in a microfluidic device made up of multiple parallel and straight microchannels^26^. Each microchannel has a uniform rectangular cross section coupled to a smaller constriction at the outlet where a large particle in the suspension initiates the clog in a stochastic manner. Without the smaller outlet constriction, however, flowrate decays linearly in a single straight microchannel where adhesive interactions exist between the particles and the constriction wall^18^.

The clogging mechanisms of rigid particles in non-tapered microchannels, and similar geometries designed to mimic structures of specific pore spaces, seem to be well established^1^. However, less is known about clogging in tapered geometries, where flow converges or diverges as the constriction progressively narrows or widens in the direction of flow^28,32^.

A converging tapered geometry can simulate flow behavior in a channel where the cross-sectional area changes over multiple orders of magnitude or length scales such as found in arterial blood circulation^33^ and multistage filtration systems^34^. It is also found in 3D printer nozzles^35,36^, and often used to model blood flow through stenosed arteries^37,38^.

The flow of suspensions in tapered geometries is a complex phenomenon as it results from a combination of the suspended particles properties, hydrodynamics, and geometric factors^1^. Several simulation studies focus on the impact of stenosis, or narrowing of vessels, on the flow of blood. In this context, the distribution of wall shear stresses and hydraulic resistance^39^, have been measured, with channel taper angle^37,39^, fluid velocity^38,39^, and viscosity^40^, each determining flow through tapered systems. In rigid particle suspensions, clogging can be delayed in a tapered microchannel by increasing the width at the outlet, thereby creating higher shear stresses that prevent suspended particles from adhering to the constriction wall to initiate clogs^28^. It is worth noting that the lengths of the tapered systems investigated so far are considerably short as the taper angles are high.

While previous studies discuss the flow and clogging behavior of particles in tapered microchannels, they rarely examine transient properties of the process—particularly, the decrease in suspension flowrate per unit time for constant driving pressure flows or increase in driving pressure when flowrate is held constant. However, a better understanding of clogging dynamics is important in optimizing the design of tapered systems to minimize clogging^41,42^. It may also be useful in predicting the performance of multistage filtration systems for maintenance planning purposes and optimizing their design to prevent plant shutdowns^43^.

Therefore, in this study, the clogging of rigid particles in a custom-made microfluidic device made up of 165 parallel and axisymmetric tapered microchannels is investigated at constant driving pressures. $w_c \approx 10 d_p$ at the inlet of each microchannel, and tapers to $w_c \approx d_p$ at the outlet. This design allows for the simultaneous observation of clogging by sieving mechanism at the channels exit and bridging in other positions along the channels. Results show the suspension flowrate decays by a power law, regardless of particle volume fraction and driving pressure. The presence of discontinuous clogging causes the flowrate to decay significantly more slowly than in straight channels. Two different particle packing behavior are observed as a function of driving pressure, and micrographs of the clogged microchannels reveal clogs formed both by sieving and bridging in each microchannel.

## Results

**Figure 1:**
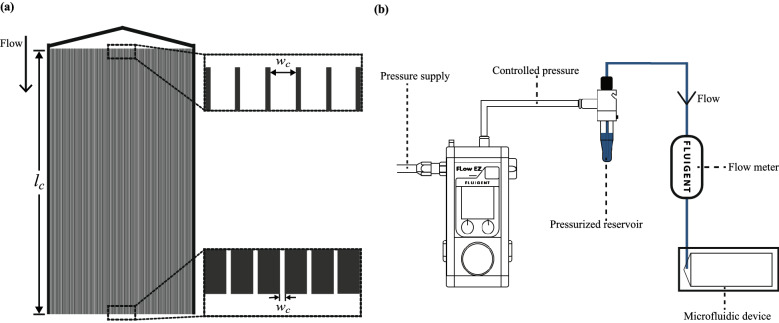
(**a**) Schematic of the microfluidic device with an inlet reservoir and 165 parallel microchannels. The channels are $\sim$ 5 cm long and taper from 40 μm at the inlet to 4 μm at the outlet, with a taper angle of $\sim 0.02^{\circ }$. The insets show the channel width at inlet and outlet; the outlet region is where the evolution of fluorescence intensity is monitored over time. (**b**) Flow test setup: a constant pressure is applied to the reservoir containing the suspension by a clean air supply. The flowrate is measured per unit time by an inline flow meter and the data is collected online. The driving pressure is approximately equal to the pressure drop across system since the fluid exiting the microfluidic device is at atmospheric pressure.

As illustrated in Fig. 1, the decay of suspension flowrate (*Q*) through a system of parallel tapered microchannels is studied at constant driving pressures ($\Delta P$) and particle volume fractions ($\phi$). Each microchannel has a constant depth ($d_c \approx 10\,$μm), but the width tapers from the inlet to the outlet at an angle of $\sim 0.02^{\circ }$, over a length ($l_c$) of $\sim$ 5 cm. *Q* for each condition of $\Delta P$ and $\phi$ is measured every second.

### Effect of particle volume fraction

**Figure 2:**
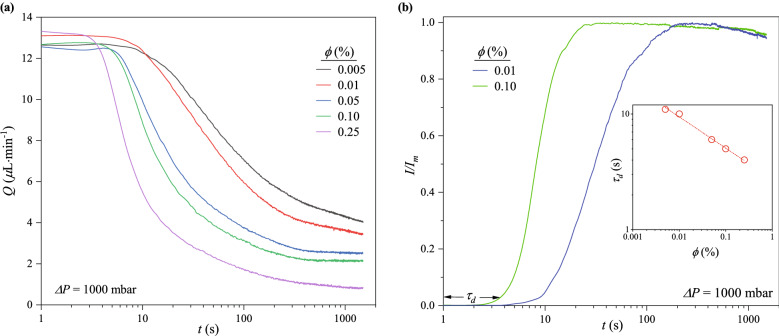
(**a**) Suspension flowrate decays over time at different $\phi$ for $\Delta P = 1000$ mbar. Even at high $\phi$, the devices never fully clog due to continued filtration of the suspension medium through the clogs. Three specific processes are present in each decay curve. They correspond to the initial and final plateau, and the power-law decay between them. (**b**) Evolution of the normalized fluorescence intensity ($I/I_m$) around the midpoint of the channels exit region for $\Delta P$ = 1000 mbar. $I/I_m$ increases as particles begin to sieve into the channels where $w_c \approx d_p$. The time, *t*, when $I/I_m$ significantly increases corresponds to $\tau _d$, which denotes the end of the initial plateau in the flowrate decay curves. Inset: $\tau _d$ depends on $\phi$ with a power-law exponent of $-\frac{1}{4}$. $\tau _d$ of the least concentrated cases ($\phi = 0.005\%$ and $0.01\%$) is approximately equal to $\tau _v$. The dip in $I/I_m$ at longer times is due to photo-bleaching arising from prolonged exposure to the excitation light source.

Presented in Fig. 2a are the flowrate decay curves at $\Delta P$ = 1000 mbar for different $\phi$. They show the devices never fully clog as the flowrates decay asymptotically towards some final value which depends on $\phi$. Differences in the hydraulic resistance ($R_H$) of the microfluidic devices account for the differences in the initial flowrates observed in Fig. 2a (see Table S1 in SI).

Three distinct processes are identified in each flowrate decay curve (Fig. 2a): (1) an initial plateau; (2) followed by a rapid flowrate decay; (3) and a much slower flowrate decay or decay end, indicated by a final plateau. The duration of first two processes are described by the timescales $\tau _d$ and $\tau _f$, which represent the ends of the initial plateau and rapid decay, respectively. $\tau _v$ refers to the time required to fill the microfluidic device with pure water.

#### Initial clogging by sieving

$\tau _d$ is the time when *Q* decays by $\sim$ 5$\%$ from the initial flowrate ($Q_0$). It denotes the end of the initial plateau and start of the following rapid decay. As seen in Fig. 2a, $\tau _d$ depends on $\phi$ as it decreased from 11 s to 4 s as $\phi$ increased from 0.005 to 0.25%. Since it takes $\sim$ 10 s ($\tau _v = V_d/Q$) at $\Delta P$ = 1000 mbar to fill the volume of the microfluidic device ($V_d$) with pure water, $t \le \tau _d$ is the period when pure water is expelled from the device and replaced with the suspension. It also corresponds to the time when particles have sufficiently sieved into positions where $w_c \approx d_p$ and the residence time of a particle in the channel, $\tau _r$. At $t=0$ s, $Q=Q_0$, and $\tau _r= 165d_cl_cw_{cm}/Q_0\sim 8$ s, where 165 represents the number of channels and $w_{cm}$ the median channel width.

Sieving of particles into the channels during this period is confirmed by analyzing the evolution of particle fluorescence intensity at the midpoint of the channel exit area (Fig. 1a). From the results (Fig. 2b), significant increases in fluorescence intensity ($I/I_m$), indicating the arrival of particles in the region under view and clogging by sieving, is observed when $t \approx \tau _d$. $I/I_m$ continues to increase until the clogs in the region stopped growing and the fluorescence signal becomes saturated. The slight decreases in the two traces of $I/I_m$ beyond their maxima (Fig. 2b) likely result from photo-bleaching due to prolonged exposure to the excitation light source^44^.

Compared to more concentrated suspensions, Fig. 2a shows the flowrate decay takes a slightly longer time to commence for more dilute suspensions. This is simply because suspensions with higher $\phi$ deliver more particles per time. As illustrated in Fig. 2b (inset), $\tau _d$ exhibits a power-law relationship with $\phi$ for $\Delta P = 1000$ mbar, where ${\tau _d} \backsim {\phi }^{{-}\frac{1}{4}}$. This represents a much weaker dependence on $\phi$ than in clogging of a series of short constrictions, for instance, in which the average clogging time scales^16^ like $\phi ^{-1}$. The import of this power-law dependence is discussed further in subsequent sections.

#### Growth of clogs by successive bridging

The decay in *Q* at $\tau _d \lesssim t \lesssim \tau _f$ is associated with the growth of clogs. The end of the rapid decay, $\tau _f$ is either the decay end time where a final plateau is reached or start time of the slower decay in cases where a final plateau is not reached. From Fig. 2a, a final plateau is achieved for all $\phi$ except the most dilute cases, 0.005% and 0.01%.

**Figure 3:**
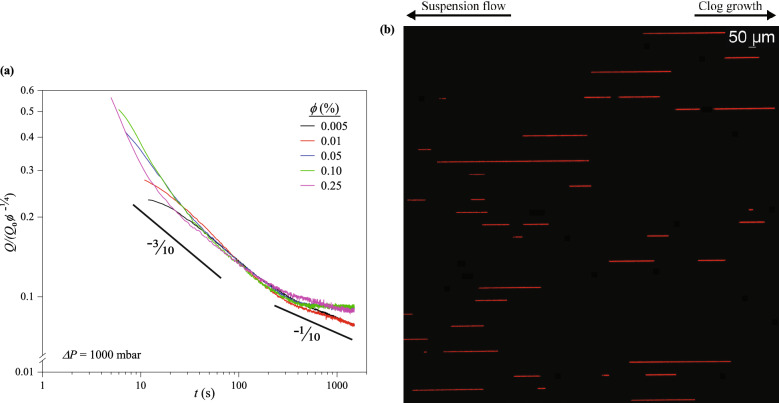
(**a**) Rescaling of flowrate decay curves for $t \ge \tau _d$ and all $\phi$ when $\Delta P$ = 1000 mbar. The flowrate decays collapse into a single master curve in the timescale $\tau _d \lesssim t \lesssim \tau _f$, indicating $\phi$ only affects the clogging rate and $\widetilde{Q} \backsim t^{-\frac{3}{10}}$. For $t \gtrsim \tau _f$, the decay curves do not follow a single scaling: a final plateau is reached for more concentrated suspensions ($\phi$ = 0.05, 0.10 and 0.25%) but a second power-law decay with an exponent of $\frac{1}{10}$ is observed for $\phi = 0.005$ and $0.01\%$, beyond the point of inflection. (**b**) Micrograph of clogged channels showing the discontinuity of clog growth in a system of parallel tapered microchannels. Multiple distinct clogs (fluorescent red streaks or spots) of different lengths are formed in different positions in some of the channels under view.

At $\tau _d \lesssim t \lesssim \tau _f$, the flowrate decays collapse onto a single curve (Fig. 3a) when *Q* is divided by ${Q_0} \phi ^{-\frac{1}{4}}$. The rescaled $\widetilde{Q}$ is fit by a power-law function:

1 $$\begin{aligned} \widetilde{Q} \equiv \frac{Q}{{Q_0}} \backsim \phi ^{-\frac{1}{4}} t^{-\frac{3}{10}} \end{aligned}$$

Barring differences in $\tau _d$, the collapse of the flowrate decays into a single curve (Fig. 3a) suggests $\phi$ impacts the magnitude of clogging only, and not necessarily the clogging mechanism^18^. Since the channels are of finite volume, there is a maximum number of particles that can effectively pack into them, regardless of $\phi$. Therefore, as observed in Fig. 2a, the differences in the rate of flow reduction as $\phi$ is varied at constant $\Delta P$ is attributable to differences in the number of particles delivered to the channels per unit time. The results suggest the threshold for “full” clogging is attained faster as $\phi$ is increased.

Clogs grow as more particles are delivered to the channels, following the initial clogging at $w_c \approx d_p$, which results in rapid decrease in the suspension flowrate, *Q*. Scaling behavior for the rate of clog growth can be obtained from a macroscopic interfacial flux balance. The flux balance describes the velocity of the front of the clog growing upstream, which, at steady or quasi-steady state, is balanced by the velocity of the fluid, ${\overline{Q}}/A$. The steady-state flux balance performed on a single growing clog at its interface with the suspension in a non-tapered straight channel is given by:

2 $$\begin{aligned} {\overline{Q}} = \frac{A L (\phi _{clog} - \phi )}{\phi \tau _g } \end{aligned}$$

where *A* and $\phi _{clog}$ are the flow cross-sectional area and the volume fraction of particles within the cake, respectively. $\tau _g$ is the timescale associated with clog growth. Equation (2) assumes the convective process is dominant compared to any diffusive processes within the cake. It shows the steady-state flowrate (${\overline{Q}}$) is inversely proportional to $\phi$ (${\overline{Q}} \backsim \phi ^{-1}$), for a clog that grows a length *L* within the time $\tau _g$. Also, ${\overline{Q}} \backsim \tau _g^{-1}$ for constant $\phi$. However, from Eq. (1), the current study shows $\widetilde{Q} \backsim {\phi }^{-\frac{1}{4}}{t}^{-\frac{3}{10}}$.

The comparison between the measured $\widetilde{Q} \backsim {t}^{-\frac{3}{10}}$ and the predicted ${\overline{Q}} \backsim \tau _g^{-1}$ shows the flowrate decay in non-tapered channels occurs much faster than in tapered channels. The comparison further implies that, *Q* in a tapered channel depends less strongly on $\phi$ compared to *Q* in a non-tapered channel.

Both of these comparative differences from clog growth in non-tapered channels can be explained by appealing to images of the clogs in the tapered channels. Unlike non-tapered channels^15,26^, micrographs of the channels taken after each flow test reveal clogs do not grow continuously from the initial points. Instead, new clogs with different number of particles, $N_c$, in their cross section are formed in succession and in random positions where $w_c > d_p$, along $l_c$. The particles form arches across the channels and $N_c \backsim \frac{w_c}{d_p}$. This phenomenon, here regarded as the “discontinuity of clogs”, appears in all the channels, and for all $\phi$ and $\Delta P$ examined.

As demonstrated in Fig. 3a, clogs do not grow from their start points indefinitely. Rather, the formation of clogs upstream truncates the growth of any previously formed clog downstream, and the presence of upstream clogs prevents additional particles from reaching downstream clogs (Fig. 3b). Discontinuous clogs have an effective length that is shorter than if the entire channel was clogged from the start of the first clog to the end of the last clog. This discontinuity in clog growth prevents the flowrate from decaying as strongly as it would if the clogs grow continuously, and thus the decrease in *Q* has a weaker dependence on both $\phi$ and *t*.

#### Cake filtration

At $t \gtrsim \tau _f$, the clogs continue to buildup upstream, as more particles pack into the channels, without substantial decrease in flowrate. Figure 2a shows a final plateau was reached for $\phi$ = 0.05, 0.10 and $0.25\%$. The final plateau signifies the end of the flowrate decay despite continued filtration of the suspension medium through interstices in the clogs. However, for $\phi$ = 0.005 and $0.01\%$, a second power-law decay, where $\widetilde{Q} \backsim t^{-\frac{1}{10}}$, is established for the region beyond the point of inflection of the decay curves. Comparison of this second power-law exponent with that in Eq. (1), confirms a significant decrease in the rate of flowrate decay.

From Fig. 3a, $\tau _f$ is located near the point of inflection of the rescaled decay curves. $\tau _f \approx$ 400 s, and shows no strong dependence on $\phi$. Beyond $\tau _f$, the flowrate decay either reaches a plateau or follows a second power law, with exponents given in Table S1. Despite the described differences in clogging rate, it is noteworthy that the portion of the decay curves beyond $\tau _f$ approximately rescales into a single curve (Fig. 3a). Again, changes in $\phi$ have no significant effect on the underlying clogging dynamics.

Furthermore, the final flowrates (Fig. 2a) vary because more particles are delivered per unit time and packed into the channels at relatively high $\phi$. For instance, when $\Delta P$ is held constant, the number of particles delivered to the channels for $\phi = 0.25\%$ would be 50 times more than $0.005\%$. Thus, it appears a high $\phi$ increases the clogging rate due to the supply of more particles and formation of more clogs (see Fig. S4 in SI).

### Effect of driving pressure

#### Suspension flowrate decay

**Figure 4:**
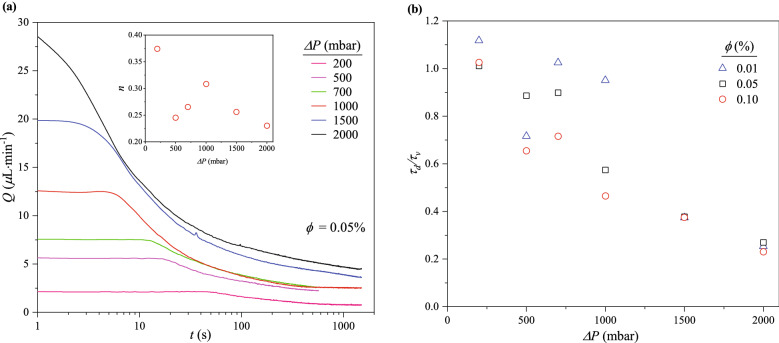
(**a**) Suspension flowrate decays over time at different $\Delta P$ for $\phi = 0.05\%$. Three processes including clogging by sieving, clog growth, and cake filtration are identified. Inset: Exponent, *n*, of the power-law decay associated with the clog growth subprocess as a function of $\Delta P$. (**b**) Ratio of decay start time ($\tau _d$) to time required to fill the microfluidic device with pure water ($\tau _v$) as a function of $\Delta P$. For each $\phi$, $\tau _d/\tau _v \approx 1$ at relatively low $\Delta P$, but decreases as $\Delta P$ increases.

The flowrate decay curves for various $\Delta P$, with $\phi$ fixed at 0.05%, are presented in Fig. 4a (see Fig. S5 in SI for $\phi$ = 0.01 and 0.10%). Three processes congruous with observations at constant $\Delta P$ (Fig. 2a) are also identified: a power-law decay is sandwiched between an initial and final plateau. Figure 4a (inset) shows the power law exponent relating to clog growth, *n*, generally decreases with $\Delta P$. The dependence on $\Delta P$ is greater than the run-to-run variation in *n* ($\sim 8$%; see Fig. S2 in SI). Interestingly, this overall trend holds true for the more concentrated $\phi =0.10\%$, while for the more dilute $\phi =0.01\%$, *n* generally increases with $\Delta P$ (see Fig. S5 in SI). It is worth noting that a final plateau in *Q*(*t*), beyond the points of inflection of the decay curves, was not reached for $\Delta P$ = 1500 and 2000 mbar even though the flow tests were stopped just before the cakes began growing into the inlet reservoir of the microfluidic device. This happened at $t \approx$ 600 s for $\Delta P$ = 500 and 700 mbar—hence the short length of their respective decay curves.

The end of the initial plateau also signifies the start of the flowrate decay and corresponds to the timescale ($t \lesssim \tau _d$) when particles sieve into the channels at $w_c \approx d_p$. Although both the time to fill the microfluidic device with pure water ($\tau _v$) and the particle residence time ($\tau _r$) are inversely proportional to $\Delta P$, it was observed that $\tau _d \approx \tau _v$ for the lowest $\Delta P$ (200 mbar) but $\tau _d < \tau _v$ when $\Delta P$ is increased. As seen in Fig. 4b, $\tau _d$ decreases to a fraction of $\tau _v$ as $\Delta P$ increases to 2000 mbar. The decrease in $\tau _d/\tau _v$ from $\sim$ 1 to $\sim$ 0.3 does not depend strongly on $\phi$, particularly for $\Delta P$ > 1000 mbar. The differences between $\tau _d$ and $\tau _v$ can be attributed to increased delivery rate of particles to the channels at relatively higher $\Delta P$. This difference in $\tau _d$ and $\tau _v$ is also evident in the evolution of fluorescence intensity at the channel exit region (see Fig. S6 in SI). The progressive decrease in $\tau _d/\tau _v$ suggests the number of particles required to reduce the suspension flowrate, *Q*, by $\sim$ 5% is delivered to the channels at a faster rate as $\Delta P$ increases.

On the other hand, there appears to be a dependence of $\tau _d/\tau _v$ on $\phi$ for $\Delta P \le$ 1000 mbar as the highest $\tau _d/\tau _v$ is observed when $\phi$ = 0.01% (Fig. 4b). This is because the number of particles that will decrease *Q* by $\sim$ 5% would be delivered at a much slower rate when both $\phi$ and $\Delta P$ is low. However, at $\Delta P>$ 1000 mbar, the high pressure forces dominate any effects of $\phi$.

The second process, occurring at $\tau _d \lesssim t \lesssim \tau _f$, is a power-law decay associated with cake growth due to successive bridging events in the channels at positions where $w_c > d_p$. Unlike the results in Fig. 3a, the flowrate decay curves do not rescale into a single curve. Here, $Q \backsim t^{-n}$ while *n* varies in the range 0.2–0.4 and depends on $\Delta P$ (see Table S1 in SI for a complete list of the power-law decay exponents for all conditions of $\Delta P$ and $\phi$).

Similar to the flowrate decay curves in Fig. 2a, the third process ($t \gtrsim \tau _f$) also begins at $t \approx$ 400 s for all $\phi$ and $\Delta P$ studied. Beyond $\tau _f$, the clogs grow as particles continue to pack into the microchannels. In most cases, the flowrate reaches a final plateau. In some other cases, however, it decays to a second power law with exponents given in Table S1 in SI. Summarily, no significant flowrate decay is observed at $t \gtrsim \tau _f$ as the suspension medium filters through the voids in the clogs.

#### Particle packing in the channels

The final suspension flowrates ($Q_f$) also differ based on $\Delta P$ (Fig. 4a), which suggests significant particle packing differences in the channels. To characterize this, the permeability, packing density, and area coverage of the filter cakes in the channels, and number of clogs were evaluated as a function of $\Delta P$ at different $\phi$.

**Permeability.** To assess how permeable the clogged channels are to the flow of the suspension medium, $Q_f$ at different $\Delta P$ and $\phi$ were fitted to Darcy’s Law:

3 $$\begin{aligned} \frac{\mu Q_f l_c}{A} = {\kappa }{\Delta P} \end{aligned}$$

where $\mu$, $\kappa$ and *A* are the suspension viscosity, Darcy’s permeability constant and cross-sectional flow area, respectively. Here, $Q_f$ is the average flowrate of the final 60 s in the decay curve.

**Figure 5:**
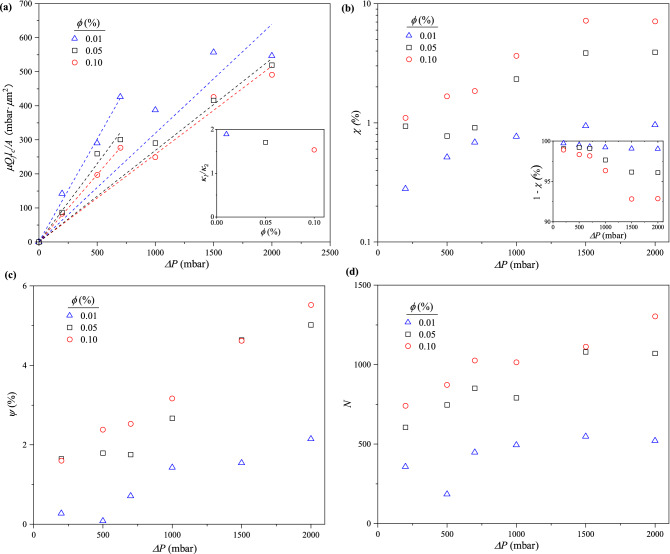
The presence of two $\Delta P$ regions (one below and above $\Delta P = 1000$ mbar), indicating significant differences in particle packing in the channels after “full clogging” is established. This is illustrated by (**a**) Device permeability ($\kappa$) estimated by fitting the final flowrate ($Q_f$) to Darcy’s Law. Inset: the ratio of $\kappa$ for $\Delta P < 1000$ mbar region to $\Delta P \ge 1000$ mbar ($\kappa _1/\kappa _2$) slightly decreased as $\phi$ was increased. (**b**) Percentage of device volume occupied by particles ($\chi$). Inset: percentage of voids in the clogged device (porosity). (**c**) Coverage ($\psi$) is the percentage of the total channel area filled with clogs. (**d**) Number of clogging events (*N*). In all cases of $\Delta P$ and $\phi$, *N* is greater than the total number of channels.

As illustrated in Fig. 5a, two distinct values of $\kappa$ become evident for each $\phi$, upon fixing a no-flow condition at $\Delta P$ = 0 mbar. Each $\kappa$ is associated with a different $\Delta P$ region for all $\phi$: one below and above $\Delta P$ = 1000 mbar. $\kappa$ for the region where $\Delta P \ge 1000$ mbar ($\kappa _2$) is lower than that for $\Delta P < 1000$ mbar ($\kappa _1$). For instance, when $\phi = 0.01$ %, $\kappa$ decreases from 0.60 μm$^2$ at low $\Delta P$ to 0.32 μm$^2$ at high $\Delta P$ (see Table S1 in SI for other values). This reduction in permeability at high $\Delta P$ is likely due to closer particle packing resulting from increase in pressure forces acting on the particles, and the presence of more particles.

It is also evident in Fig. 5a that $\kappa$ increased with decreasing $\phi$, for the two $\Delta P$ regions. A comparison of the ratio of $\kappa _1$ to $\kappa _2$ for each $\phi$, presented in Fig. 5a (inset), shows the clogged channels are generally less permeable to the filtrate as $\phi$ increases: $\kappa _1/\kappa _2 \approx 1.9$ at $\phi = 0.01$%, decreasing to $\sim$ 1.5 at 0.10 %. This can also be attributed to the delivery of a higher number of particles to the device per unit time, at higher $\phi$ conditions. Moreover, the presence of more particles would result in increased number of clogging events, which would provide higher resistance to the flow of the suspension medium (see Fig. S4 in SI).

**Packing density.** This is the percentage of the total volume of the channels occupied by particles after “full clogging.” For each condition of $\phi$ and $\Delta P$, the flowrate decay is integrated over the duration of the flow test to determine the volume of suspension that flowed through the device, and hence, the total volume of particles delivered. The estimation of packing density ($\chi$) is based on the assumption that suspended particles delivered to the device during the flow tests made it into the channels and participated in the formation and/or growth of the clogs.

4 $$\begin{aligned} \chi = {\frac{\phi }{A_c d_c}}\left[ \int _{0}^{t} Q \,dt - V_r\right] \end{aligned}$$

where $A_c$ and $d_c$ are the total channel area and depth, respectively. Equation (4) only considers the packing of particles in the channels, excluding the inlet reservoir volume, $V_r \approx 0.15\,$μL.

Changes in $\chi$ on a log scale at different $\phi$ as a function of $\Delta P$ are presented in Fig. 5b. It shows $\chi$ increases with increasing $\phi$ and $\Delta P$. Because $\chi$ is defined in terms of the entire flowrate decay curve *Q*(*t*), not $Q_f$, the two regimes seen in Fig. 5a are somewhat less evident in Fig. 5b. Rather, $\chi$ reaches a plateau at $\Delta P=1500$ mbar for each $\phi$. The linear axes of the inset shows that porosity, $1-\chi$, decreases with $\Delta P$, reaching a plateau at 1500 mbar. The magnitude of the plateau decreases as $\phi$ increases, from 99% at $\phi =0.01$% to 92% for $\phi =0.10$%.

**Coverage.** This is the fraction of the total surface area of the channels occupied by clogs. The area coverage ($\psi$) is obtained from image analyses of clog locations, and each clog is treated as a rectangular space.

5 $$\begin{aligned} \psi = \frac{1}{A_c}\sum _{i = 1}^{N}{w_i}{l_i} \end{aligned}$$

where $w_i$ and $l_i$ represent the width and length of clog *i*, respectively. *N* is the total number of clogging events.

From Fig. 5c, $\psi$ also increases as $\phi$ and $\Delta P$ increase. Only a small fraction of the channels clog: for instance, $\sim$ 6% of the entire channel area is occupied by clogs at the highest $\Delta P$ (2000 mbar) and $\phi$ (0.10%). This is in agreement with the results in Fig. 5b, which shows $\sim$ 8% of the device volume is filled with particles at the same conditions. This similarity corroborates the two types of measurements: $\chi$, the packed channel volume estimated from *Q*, and $\psi$, the areal fraction of clogs estimated from image analysis.

In addition, the particle packing density in the clogs themselves ($\phi _{clog}$) can be estimated as the ratio of the packing density in the channels ($\chi$) to the areal coverage of the clogs ($\psi$).

6 $$\begin{aligned} \phi _{clog}= \frac{\chi }{\psi } = {\frac{\phi }{d_c \sum _{i = 1}^{N}{w_i}{l_i}}}\left[ \int _{0}^{t} Q \,dt - V_r\right] \end{aligned}$$

where the numerator represents the total volume of particles delivered to the channels and the denominator is the total volume of the clogged regions. This combination of flowrate data with imaging provides estimates of $\phi _{clog}$ that are close to known values for various packing types. For $\phi$ = 0.05%, the average $\phi _{clog} \approx 0.50$ for the low $\Delta P$ regime and increases to $\sim$ 0.80 at higher $\Delta P$. This further shows that the packing of the particles in the clogs is more compact in the high $\Delta P$ region.

**Number of clogging events.** Figure 5d shows the total number of clogging events, *N*, as measured by image analysis, monotonically increases at different $\phi$ as $\Delta P$ was increased. It also reveals two $\Delta P$ regions. However, *N* varies linearly as $\phi$ at constant $\Delta P$ (see Fig. S4 in SI). The value of *N* also alludes to the occurrence of multiple clogs in each microchannel, even at low $\Delta P$ and $\phi$. There are 165 channels in the device, but the least *N* is approximately 200, growing to nearly 1500, indicating increase in the number of clogs per channel from $\sim$ 1 to 9 as both $\Delta P$ and $\phi$ increase.

The increase in *N* can in part be explained by an increase in the total number of particles delivered to the channels, $N_f$, estimated by integrating *Q* over the duration of the flow test:

7 $$\begin{aligned} N_f \approx \frac{\phi }{V_p}\left[ \int _{0}^{t} Q \,dt - V_r\right] \end{aligned}$$

where $V_p = \frac{\pi }{6}{d_p}^3$, the volume of a spherical particle. Similar to Eq. (4), the subtraction of $V_r$ is done so that $N_f$ reflects particles packed in the channels, and not in the inlet reservoir.

Comparing Eqs. (4) and (7) shows that $N_f = A_c d_c \chi / V_p \sim 4\times 10^7 \chi$. Thus, $N_f$ behaves like Fig. 5b, increasing with both $\Delta P$ and $\phi$. A further comparison of $\chi$ with *N* shows that all three quantities increase both with $\Delta P$ and $\phi$. An increase in particles delivered, $N_f$, suggests the formation of more clogs, with lengths that may depend both on $\phi$ and $\Delta P$.

The behavior seen in Fig. 5d may lend some insight into the dynamics seen in the flow tests. In particular, as seen in Fig. 3a and Table  S1, the decay in *Q*(*t*) transitions to a slower power-law decay at $t\sim \tau _f$ for the lower values of $\phi$ studied. For flow tests at higher $\phi$, *Q*(*t*) seems to reach a plateau at $t \gtrsim \tau _f$. Fig. 5d also shows that *N* increases with $\phi$. However, the increase in *N* is not proportional to the increase in $\phi$. While the data span an order of magnitude in $\phi$, *N* increases by a factor of $\sim$ 2 only. This suggests the presence of more clogs at higher $\phi$ corresponds to shorter clogs. Also, lower *N* observed in the most dilute conditions of $\phi$ suggests the clogs may be longer. Since the length of the clogs corresponds to their growth timescale, the continued growth of longer clogs may lead to the second, slower power-law decay as in Fig. 3a and Table S1 in SI. At the same time, a larger *N* of shorter clogs at higher $\phi$ suggests clogs stop growing at shorter times, thus potentially explaining the plateaus seen in *Q*(*t*) at higher values of $\phi$.

## Discussion

### Influence of channel geometry on clogging timescale and flowrate decay

$\tau _g$ from Eq. (2) is a timescale related to the growth of clogs. In different non-tapered and parallel multichannel systems, Refs.^16,45^ define $\tau _g$ as the mean interval between two clogging events. In a single non-tapered channel, it was measured as the time required for a decrease in the initial suspension flowrate by 10%^18^. Both types of experimentally measured timescales match the prediction, from the steady-state flux balance in Eq. (2), that $\tau _g \backsim {\phi }^{-1}$. However, results obtained in this study shows ${\tau _g} \backsim {\phi }^{{-}\frac{1}{4}}$, where ${\tau _g} \equiv \tau _d$ (Fig. 2b). $\tau _d$ is the end of the initial plateau in the flowrate decay (Fig. 2a) and time taken for $\sim$ 5% decrease in flowrate. The power-law exponent shows the timescale of the initial clog formation in tapered channels is faster, compared to non-tapered geometries.

In a transient case at constant $\Delta P$, *Q* decays as the clog grows until a plateau is reached. Results presented in Ref.^18^ for a single non-tapered channel shows ${Q} \backsim t^{-1}$ in the period intervening the initial clog formation and decay end time. However, as shown in Eq. (1), ${Q} \backsim t^{-\frac{3}{10}}$ for the tapered multichannel system described in the current study. Since the clogging patterns in each channel were observed to be identical and independent of other channels, the clogging of multiple channels can be approximated to be a multiplication of the observation in a single channel.

It is worth noting that $1 \lesssim \frac{w_c}{d_p} \lesssim 10$ in the current study while $\frac{w_c}{d_p} = 20$ in Ref.^18^—a value above the threshold within which clogs can be formed by bridging or sieving^1^. Clogging was thus initiated by the introduction of adhesive interactions between the particles and the constriction wall. Nonetheless, comparison with results in the current study does suggest the observed power-law decay, regardless of $\phi$ (Fig. 3a) or $\Delta P$ (Fig. 4a), is a phenomenon peculiar to the tapered geometry of microchannels that can accommodate between 1 and  10 particles across their channel width. Interestingly, the power-law decay persists even when the channel depth is increased to 22.5 μm (see Fig. S7 in SI). The power-law scaling also suggests that, though the initial clog formation time is faster, flowrate decays at a significantly slower rate in a tapered channel ($Q\sim t^{-n}$, where $n<1$) when compared to a non-tapered geometry ($Q\sim t^{-1})$.

### Discontinuity of cake growth

**Figure 6:**
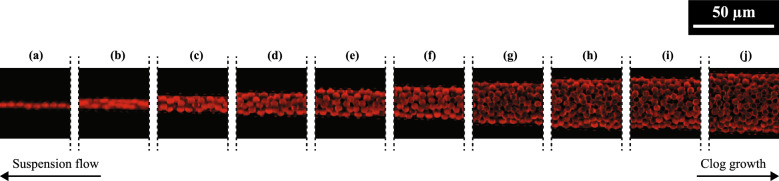
Micrographs showing sections of distinct clogs in a tapered channel, for $\Delta P$ = 2000 mbar and $\phi$ = 0.05%. None of the clogs are connected, and the suspension medium is able to filter through the voids in the packed particles. In images (**a**)–(**j**) are clogs with 1–10 particles spanning the width of the constriction, respectively. Clogs are formed by sieving where $w_c/d_p \approx 1$, and by bridging mechanism in positions where $w_c/d_p \ge 2$.

Besides the measured number of clogs being greater than the total number of channels (Fig. 5d), micrographs of the clogged channels also show multiple clogs are formed in each microchannel. In Fig. 6 are images of different sections of a clogged microchannel, for $\phi$ = 0.05% and $\Delta P$ = 2000 mbar. The micrographs show each clog has a specific number of particles, $N_c$, spanning the entire cross section of the constriction. The same pattern is observed in each microchannel, and for all conditions of $\Delta P$ and $\phi$.

The initiation of a clog upstream within any channel truncates the growth of any preceding clog, downstream in the same channel. This clog initiation, growth and truncation process occurs throughout the entire length of each channel, beginning at the outlet where $w_c \approx d_p$ and resulting in the formation of multiple distinct clogs along the length of every channel. This differs from observations in non-tapered multichannel systems where, at long timescales, single clogs formed at an initial point grow approximately the same length towards the inlet area, though the initial clog formation times may be different^26^.

The successive bridging and discontinuous clog growth observed in the tapered system described in this study is a stochastic process. It is likely that it solely results from hydrodynamic effects preventing the simultaneous passage of flowing particles through the constriction^46^. Or, as in more concentrated suspension flows, the coupling of an increasingly converging flow path and hydrodynamic factors that repeatedly increase the local $\phi$ to the point of jamming^47^.

Interestingly, discontinuous clogs occur even at the highest driving pressure ($\Delta P=2000$ mbar). From another study, it is clear that increasing flowrate, and hence driving pressure, can provide enough force to dislodge nascent clogs and enable continuous flow^47^. Furthermore, sinusoidal fluctuations in $\Delta P$ between 25% and 125% of the mean, can delay clogging^15^. These observations might suggest that flows driven at higher $\Delta P$ through tapered channels might allow continuous growth of a clog from its initial point at the channel outlet. However, as observed in our experimental results, even $\Delta P=2000$ mbar is not sufficient to break subsequent upstream clogs and allow the formation of a single, long clog in each channel.

### Effect of particle arrangement on post-clogging permeability

The observation of two distinct values of post-clogging permeability, $\kappa$, suggests differences in the packing behavior of particles in the clogged channels based on $\Delta P$, and for each $\phi$ (Fig. 5a). While images of the clogs in Fig. 6 only show a *z* position, the depth of the microchannels ($d_c \approx$ 10 μm) allows for two layers of particle packing in each clog as $d_p \approx 4~$μm. The degree of randomness of the particle packing is observed to increase as ${w_c}/{d_p}$ increases: clogs having 1–4 particles in their cross section, as seen in Fig. 6a–d, appear to be more ordered . Clogs with more particles and as many as 10 particles across the width of the channel are less orderly (Fig. 6e–j). Thus, the packing order seems to decrease as the distance from the channel exit increases.

The degree of order or disorder of the particles in the clogs may directly influence the porosity of the clogs. For instance, cubic packing of monodisperse spheres have a porosity of $\sim$ 48%^48^. However, the porosity may vary between 36–44% in random arrangements, depending on whether the particles are loosely or closely packed^48,49^. In either case, clogs constituted by randomly-packed particles have a significantly lower porosity or higher packing fraction, compared to cubic arrangements. The application of pressure forces to drive the suspension through the microchannels increases the possibility of random close particle packing, which have a porosity of $\sim$ 36%^50^.

More clogs are formed in relatively high conditions of $\Delta P$ and $\phi$, as seen in Fig. 5d. Devices used in flow tests conducted under such conditions may have more clogs containing random and closely packed particles. Indeed, the estimate of $\phi _{clog}$ suggests an increase in packing as $\Delta P$ increases. The increased presence of more tightly packed clogs in the channels will reduce the post-clogging permeability of the device, as shown in Fig. 5a^50^.

## Conclusion

The clogging of rigid particles in a microfluidic device made up of parallel tapered microchannels was investigated by analyzing the decline of suspension flowrates at constant driving pressures. Three processes which include an initial clogging by sieving, a rapid flowrate decay due to successive, discontinuous bridging, and a cake filtration process are observed for all conditions of volume fraction and driving pressure studied. The successive bridging is associated with a power-law decay of the flowrate and appears to be peculiar to the tapered geometry. Micrographs of the clogged channels confirm multiple, discontinuous clogs are formed by successive bridging in each microchannel, irrespective of the particle volume fraction and driving pressure. The results suggest it is more attributable to the tapered geometry than hydrodynamic factors as the phenomenon was observed in all microchannels even at low particle volume fraction and relatively low driving pressure.

Two regions of driving pressures are observed upon fitting the final flowrates of the suspension to Darcy’s Law for different particle volume fractions. Each region has a distinct permeability, which was found to be due to differences in the number of particles delivered to the microchannels, the number of clogs formed, and particle packing in the microchannels. More particles are delivered to the microchannels at higher driving pressures, which leads to a higher number of clogging events. Also, clogs which grow upstream toward the inlet seem to exhibit a more random packing pattern compared to the cubic packing observed in clogs with fewer particles toward the outlet. These less porous clogs upstream consequently reduce device permeability.

The results presented here reveal features of suspension flow through tapered channels not observed or reported in non-tapered channels. The qualitative difference between suspension flow through tapered and non-tapered channels may have broader implications. In particular, the measured flowrate in a long tapered channel decays with a much weaker dependence on both particle concentration and time than in non-tapered channels. This observation may be relevant to inform filter design, especially in situations where particle capture is desired, but rapid flowrate decay is not. Furthermore, the gentle taper used in this study facilitates investigation of clogging by sieving and bridging over multiple length scales simultaneously: from clogs containing one particle to bridges containing as many as 10. Further investigation into the geometry of the observed discontinuous clogs will lend more insight into the underlying statistical mechanics.

## Materials and methods

### Microfluidic device

The microfluidic device, Fig. 1a, was fabricated with polydimethylsiloxane (PDMS) using standard soft lithography techniques, and bonded onto a glass slide after plasma cleaning, which makes the internal surfaces hydrophilic. The device is made up of 165 parallel microchannels connected to a triangular reservoir at the inlet. The length ($l_c$) and depth ($d_c$) of the channels are $\sim$ 5 cm and 10 μm, respectively. Their width ($w_c$) tapers from 40 μm at the inlet to 4 μm at the outlet. The taper angle ($\theta$) is $\sim$ $0.02^{\circ }$. The choice of this taper angle maximizes the number of channels on a standard microscope glass slide (75 mm by 25 mm) while also enabling simultaneous investigation of clogging by as few as one particle to as many as 10 particles across the channel width. With $\theta \approx 0.02^{\circ }$, a decrease in channel width by one particle diameter ($\sim$ 4 μm) occurs over a length corresponding to $\sim$ $10^4$ μm particle diameters ($\sim$ 5 cm).

The device geometry mimics the form of complex confinements where the cross-sectional flow area decreases along the flow direction. Examples are found in systems as disparate as networks of pore spaces and blood circulation. Also, the particles cannot exit the channels as $w_c \approx d_p$ at the outlet, which is applicable to systems such as multistage filters, where the final stages are purposed to capture the smallest particles^43^.

### Flow tests

A schematic diagram of the experimental setup is shown in Fig. 1b. For each experiment, a Fluigent LineUp Flow EZ™ device was used to apply a constant driving pressure ($\Delta P$) to flow a suspension through the microfluidic device. For the entire duration of each experiment, the flowrate per unit time (*Q*) was measured by a flow meter (Fluigent FLOW UNIT™) positioned in series with the flow device and directly before the inlet of the microfluidic device. *Q* is measured at 1 Hz. The flow device provides a maximum $\Delta P$ of 2000 mbar, thus constraining the range explored in this study.

The flow device has a resolution of $\sim$ 0.6 mbar, and $\Delta P$ is held constant for the duration of each flow test with fluctuations <0.2%. Also, the flow meter can measure suspension flowrates in the range 0–80 μL min^-1^, with a resolution of 0.06 μL min^-1^. Measurements of *Q* in steady conditions show fluctuations <2%. For further details, see Fig. S3 in SI.

The suspensions are fluorescent polystyrene beads with $d_p=$ 4.19 ± 0.27 μm (Bangs Laboratories) dispersed in pure water (Millipore Milli-Q) at volume fractions ($\phi$) in the range $[0.005\%, 0.25\%]$, which is 2–4 orders of magnitude below jamming volume fraction^47^. The particles are monodisperse, and stable in the suspension for the duration of each experiment. The density of the particles ($\rho _p$) is $\sim$ 1.06 g cm$^{-3}$, which is approximately the same as that of the dispersion medium (water). Hence, sedimentation is negligible over the timescale of the experiments. Also, the particles show no attractions to the PDMS channel walls, which implies that only steric effects are involved in the clogging process.

It is necessary to ensure the microfluidic devices are in proper condition before conducting the flow test. Therefore, pure water is first flowed through each device at $0>$ $\Delta P$ $\le$ 2000 mbar and the corresponding flowrates measured. Based on Hagen–Poiseuille approximation for steady laminar flow of a Newtonian fluid in a cylindrical pipe, the hydraulic resistance ($R_H$) of the device is the reciprocal of the slope of *Q* vs. $\Delta P$^51^. For each device used in this study, the plot of *Q* vs. $\Delta P$ is linear over the range of $\Delta P$ considered, with $R_H \approx$ 80 ± 5 mbar min μL^-1^ (see Fig. S1 in SI). It shows the devices do not deform even at relatively high $\Delta P$ (see Fig. S1 in SI). This procedure is necessary as the elastic deformation of PDMS under high $\Delta P$ allows more flow, which is undesirable in the current application^52,53^. In particular, the linear behavior of *Q* vs. $\Delta P$ suggests the narrower PDMS walls near the channel inlets can withstand pressure without deforming. The total volume of the device, $V_d \approx 1.8\,$μL, summing the volume of the channels and inlet reservoir.

Even at the highest $\Delta P$, the flow remains laminar in each microchannel with a Reynolds number, $Re = {u \rho L}/{\mu } < 0.5$. Where *u*, *L*, $\rho$ and $\mu$ are the flow velocity in an unclogged channel, width of the microchannel, suspension density and viscosity, respectively. The Péclet number, $Pe = {u L}/{D} \sim 10^4$, for the lowest hydrodynamic conditions. It shows convective forces dominates the clog formation and growth processes. *D* is the mass diffusivity of the particle, and was estimated using Stokes–Einstein equation: $D = {k_B T}/{3 \pi \mu d_p}$, where $k_B$ and *T* are Boltzmann constant and temperature, respectively.

All the flow tests were conducted at room temperature. Flow tests are typically stopped at *t* = 1500 s or just before the clogs grow out of the channels and particles begin packing in the inlet reservoir of the microfluidic device, which is usually visible to the eyes. In addition, new microfluidic devices are prepared for each flow test, and repeatable results are obtained despite device-to-device variations in hydraulic resistance ($R_H$). Flowrate decay curves obtained in flow tests conducted under the same conditions of $\Delta P$ and $\phi$, but with different devices, follow the same trend (see Fig. S2 in SI). The flowrates also decay to a similar final value.

### Image analyses

#### Fluorescence intensity of clogs under flow

To measure the evolution of particles fluorescence intensity at the exit, channels were imaged during some flow experiments using a Leica DMi8 microscope. The midpoint of the channel exit area was selected for imaging as the highest field of view of the microscope is much smaller than the width of the device. The images have a dimension of $\sim$ 2.5 mm by $\sim$ 2.5 mm, and were taken every second from the start of the flow experiment until the end (1500 s). Each image covers $\sim$ 30 channels, extending inwards from the exit point where $w_c \approx d_p$. The highlighted region at the device outlet in Fig. 1a indicates the location of these images with respect to the entire device.

The images were analysed using a custom routine in MATLAB. The elements in each 2048-by-2048 pixels image array were summed and subtracted from the value returned for the image taken at $t = 0$ s. The result for each image is regarded as the “fluorescence intensity” (*I*), and was further normalized by dividing by the maximum in the series ($I_m$). This was repeated for each flow experiments completed with simultaneous imaging.

#### Estimation of clogs width and length

The devices are also imaged after each flow test to measure the dimensions of the clogs. The entire channels area is divided into images covering $\sim$ 2.5 mm by $\sim$ 2.5 mm area. These images of clogged channels, obtained at different $\Delta P$ and $\phi$, are later merged into a single image and preprocessed to eliminate background noises as reasonably possible.

Since the particles are fluorescent, the image analysis algorithm was applied to identify the centroids of bright spots or streaks (clogs) in the images. The length and width of the clogs were determined as the length of the major and minor axes of the bright spots, respectively.

The lighting condition, image illumination and magnification were kept the same for all conditions of $\Delta P$ and $\phi$ studied—for easy comparison. .

## Supplementary Information

Supplementary Information.

## Data Availability

The datasets used and/or analyzed during the current study are available from the corresponding author upon reasonable request.
