# Supplementary material for: Flow dynamics through discontinuous clogs of rigid particles in tapered microchannels
Source: Sci Rep. 2022 Dec 30;12:22587. doi: 10.1038/s41598-022-25831-w (PMC9803713; doi:10.1038/s41598-022-25831-w)
Supplement: Supplementary file 1 — Supplementary Information. [file 41598_2022_25831_MOESM1_ESM.pdf]

Supporting material for: Flow dynamics through discontinuous clogs of rigid particles in tapered microchannels

Olukayode T. Majekodunmi[1] **and Sara M. Hashmi**[1,2,3,*]

[1]Department of Chemical Engineering, Northeastern University, Boston, MA 02115
[2]Department of Mechanical and Industrial Engineering, Northeastern University, Boston, MA 02115
[3]Department of Chemistry and Chemical Biology, Northeastern University, Boston, MA 02115
[*]s.hashmi@northeastern.edu

Hydraulic Resistance of the Microfluidic Device

The hydraulic resistance, R_H, was estimated by flowing pure water through each microfluidic device at driving pressures, ΔP, in the range 0 – 2000 mbar. This was done after the device had first been filled with pure water. ΔP was increased stepwise and the respective flowrates were recorded. As illustrated in Fig. S1, the results were fitted to Hagen-Poiseuille's law for pressure-driven flow in a cylindrical pipe which is simplified as $Q = \Delta P/R_H$.

R_H is constant for any device, provided the geometric parameters and fluid conditions are the same. The mean R_H of the devices used in this study was estimated to be $\sim$80 mbar·min·μL^{-1} with a standard deviation of $\sim$5 mbar·min·μL^{-1} (see Table S1 for the respective values of each device). Also, the devices were not deforming to allow more flow under high ΔP as demonstrated by the linearity of the Q *vs.* ΔP plots over the range of ΔP explored in this study.

By considering the inlet reservoir and channels region of the microfluidic device as two fluidic resistors in series, the greatest hydraulic resistance appears to be in the channel region as the R_H of the device (inlet reservoir and channel region combined) is, at least, an order of magnitude more than that of the inlet reservoir (Fig. S1).

Repeatability of Flow Tests

Although new microfluidic devices are prepared for each flow test, device-to-device variation in hydraulic resistance (R_H) does not impact the consistency of the results. Presented in Fig. S2 are flowrate decay curves obtained for different conditions of ϕ when ΔP = 1000 mbar. Within each of the three plots shown of the raw data, in Fig. S2a-c, the difference in R_H between the two trials is reflected in differences in Q_0: $Q_0 R_H = \Delta P$ in each case. For each condition of ϕ and ΔP, the two normalized flowrate decay curves follow the same trend and decay to a similar final value (Fig. S2d-f). The comparison shows the experiments are repeatable, as the decay start (τ_d) and end times (τ_f), and power-law decay exponent (n) are approximately the same in the compared cases, with n varying $\approx$ 8%. Consequently, the result of only one flow test is reported and included in the subsequent analyses, for each condition of ϕ and ΔP investigated. $R_H \approx$ 80 mbar·min·μL^{-1} for the flow tests reported in the manuscript.

Uncertainty Analysis

Fig. S3a shows the time series plot of different ΔP during the flow tests for ϕ = 0.05%. It shows ΔP is constant the entire duration of the experiments. Given the values of standard deviation (σ) indicated on Fig. S3a, the fluctuations in ΔP range from $<$ 0.2% at 200 mbar to as low as 0.005% at 2000 mbar. According to the manufacturer, the device can provide a maximum ΔP of 2000 mbar, with a resolution of $\sim$0.6 mbar and stability of 0.1% when $\Delta P \gtrsim$ 200 mbar.

Also, the flow meter can accurately measure water flowrates in the range 0 – 80 μL·min^{-1} with a resolution of 0.06 μL·min^{-1}. The accuracy is 5% of the measured value above 2.4 μL·min^{-1} and 0.12 μL·min^{-1} for values below 2.4 μL·min^{-1}. In the flow tests conducted in this study, the minimum and maximum flowrates are $\sim$1 μL·min^{-1} and $\sim$30 μL·min^{-1} respectively, which are well within the limits of the flow meter. Fig. S3b shows the variability of flowrate measurements, beyond the decay end time (τ_f) for flow tests conducted at ΔP and ϕ = 0.05, 0.10 and 0.25%. These are examples of cases where a final plateau was reached. The fluctuations in Q are no more than 2%, despite the low value of Q itself. When $Q(t)$ features a plateau at early times in the flow tests, fluctuations in Q are less than 1%.

Number of Clogging Events

Fig. S4 shows the number of clogging events, N, varies linearly as ϕ for constant ΔP. Also, it suggests there is a minimum number of clogs required for the device to be ”fully clogged" - which is ~500 in these cases. By extension, it implies a minimum number of particles is also required for the device to be "fully clogged."

Suspension Flowrate Decay

Presented in Fig. S5 is the flowrate decay curves at different ΔP when ϕ = 0.01 and 0.10%. It shows the flowrate decay is faster at ϕ = 0.10% for each ΔP. Also, each decay curve features three distinct timescales: a power-law flowrate decay preceded by an initial plateau which ends at $t = \tau_d$, and succeeded by a final plateau or slower decay rate depending on ϕ. A final plateau is reached at $t \equiv \tau_f \approx 400$ s when ϕ = 0.10%. It signifies the end of the flowrate decay. However, a final plateau is not observed at ϕ = 0.01%: instead, the cake continued to grow but at a much slower rate.

Evolution of Fluorescence Intensity

Changes in the intensity of fluorescence signals (I/I_m) at the midpoint of the channel exit area were analyzed for some flow tests to estimate the end of the initial plateau (τ_d) in the flowrate decay curves. The result when ΔP = 2000 mbar and ϕ = 0.05% is presented in Fig. S6. It indicates significant increases in I/I_m in the first second of the flow test, which is less than the time it takes to fill the device with pure water ($\tau_v = V_d/Q$) at the same ΔP: $\tau_v = V_d R_H/\Delta P$, where V_d is the total volume of the device calculated to be ~2 μL. In this case, $\tau_v \approx 5$ s while $\tau_d \approx 1$ s.

Effect of Microchannel Depth

Although the result presented in this study is based on 10 μm deep microchannels, it is important to investigate any potential change in flow dynamics and clogging pattern when the channel depth is changed.

Fig. S7 shows the results when a suspension with ϕ = 0.10% is flowed at ΔP = 500 mbar through a device with 22.5 μm deep channels. The R_H of the device is ~25 mbar·min·μL^{-1}. This value is ~3 times less than that of devices with 10 μm deep microchannels (~80 mbar·min·μL^{-1}). Fig. S5(b) includes a flowrate decay curve at the same conditions, $\phi = 0.10\%$ flowed at $\Delta P = 500$ mbar, in a device with 10 μm deep channels, where $Q_0 \approx 6$ μL·min^{-1}. In the 22.5 μm channel, $Q_0 \approx 16$ μL·min^{-1}, ~3 times greater, because R_H is ~3 times less in the deeper channels. Despite this difference in R_H, however, similar flow dynamics are observed compared to results obtained with ϕ = 0.10% and ΔP = 500 mbar in 10 μm deep channels. The initial plateau persists until $\tau_d \sim$ 10s, corresponding to the clogging by sieving step. A power-law decay, corresponding to the cake growth, is observed after the initial plateau (Fig. S7a). The exponent, n, of the power-law decay is ~0.2, which is within ~30% of that reported in the devices with 10 μm deep channels (Table S1).

A notable difference in the flow dynamics between the 22.5 μm and 10 μm deep channels is the delay in the onset of the final plateau. For 22.5 μm deep channels, no plateau is observed even up to 1500 s, which is due to lower R_H and more filtrate flow through the interstices in the clogs.

Micrographs of the clogged 22.5 μm deep channels show the clogging pattern is similar to the observation in 10 μm deep channels. The clogs grow discontinuously from the initial points, beginning where $w_c \approx d_p$, towards the channel inlet: multiple distinct clogs are observed in each channel (Fig. S7b). Unlike the 10 μm deep channels where only two layers of particles can fit within the depth of the channels, up to five layers of particles can fit in the 22.5 μm deep channels.

In short, both the clogging behavior and flowrate decay are robust despite changes in the depth of the channels. This result further strengthens the conclusion that the power-law decay is peculiar to the tapered geometry and the presence of discontinuous clogging.

Decay Timescales and Power-law Exponents

The timescales and exponents of the power-law fits observed both when the decay curve reaches a final plateau and otherwise are presented in Table S1 for all conditions of ΔP and ϕ examined in this study.

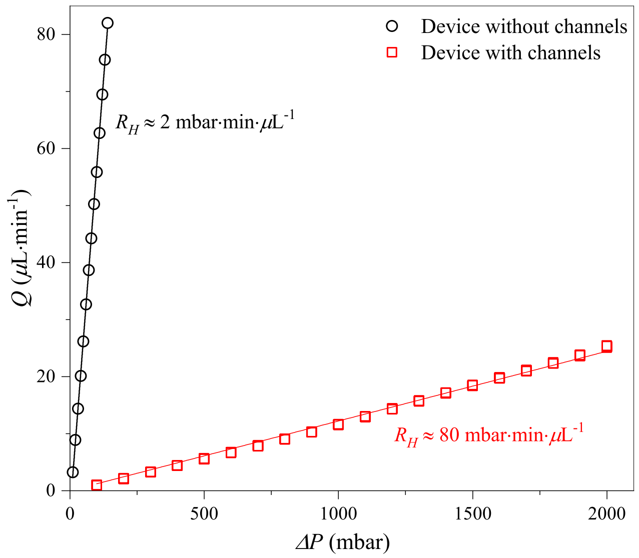

Figure S1. Linear ramp of ΔP with pure water to estimate R_H of the microfluidic device (inlet reservoir and channels combined) and inlet reservoir (device without channels).

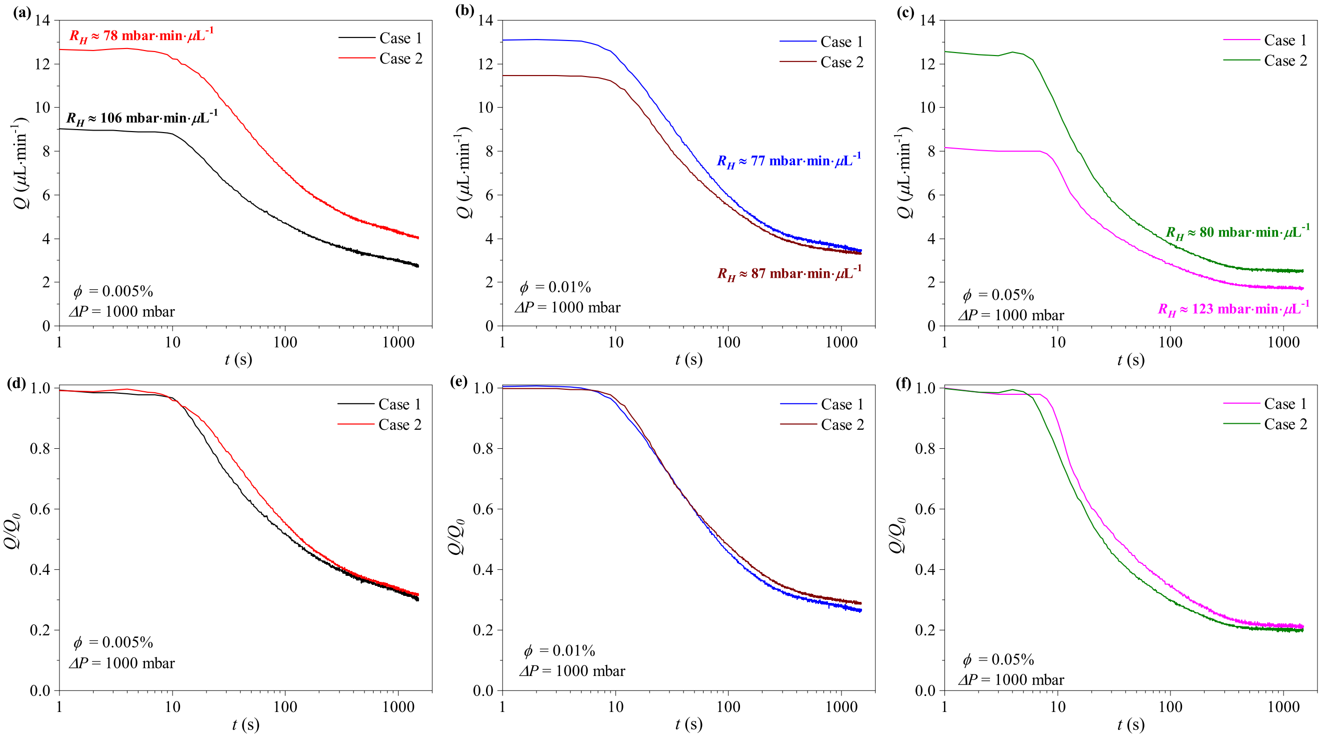

Figure S2. Comparison of suspension flowrate decay curves obtained using two different microfluidic devices for the same condition of ΔP = 1000 mbar and ϕ = **(a)** 0.005%, **(b)** 0.01%, and **(c)** 0.05%. **(d)** – **(f)** are the respective normalized flowrate decay curves (Q/Q_0).

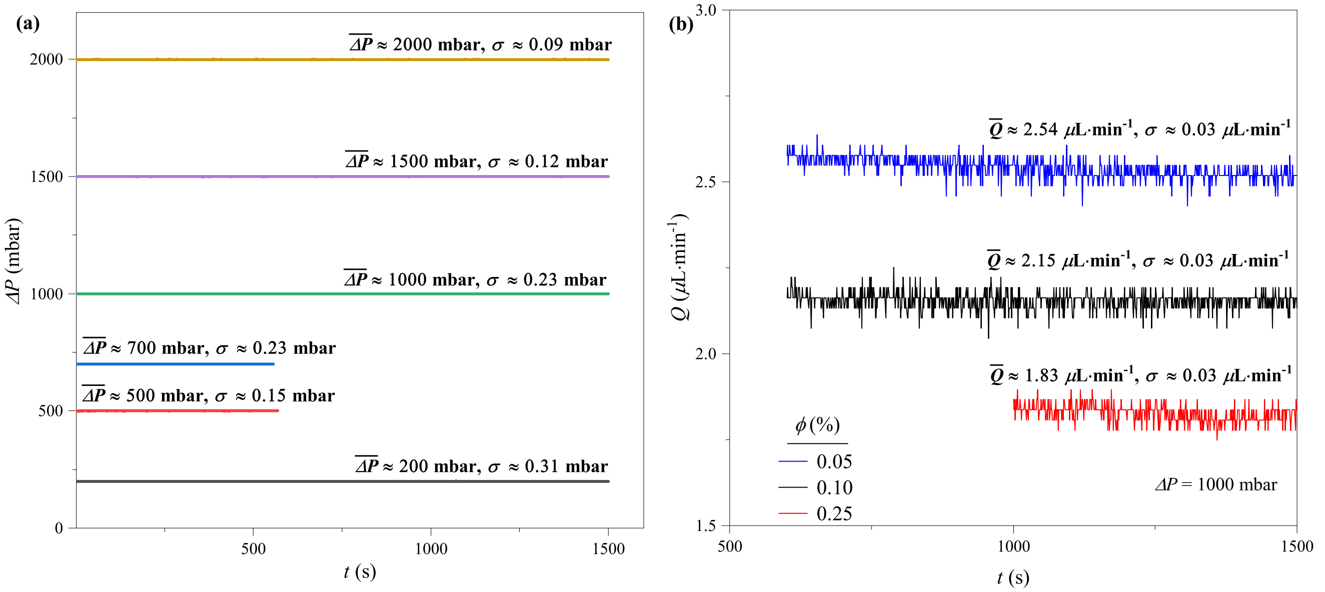

Figure S3. Variability of ΔP and Q measurements: **(a)** Stability of ΔP during flow tests for $\phi = 0.05\%$. **(b)** Variations in Q beyond the decay end time and start of the cake filtration subprocess (τ_f), a period where the flowrate is steady, for $\Delta P = 1000$ mbar and $\phi = 0.05$, 0.10 and 0.25%.

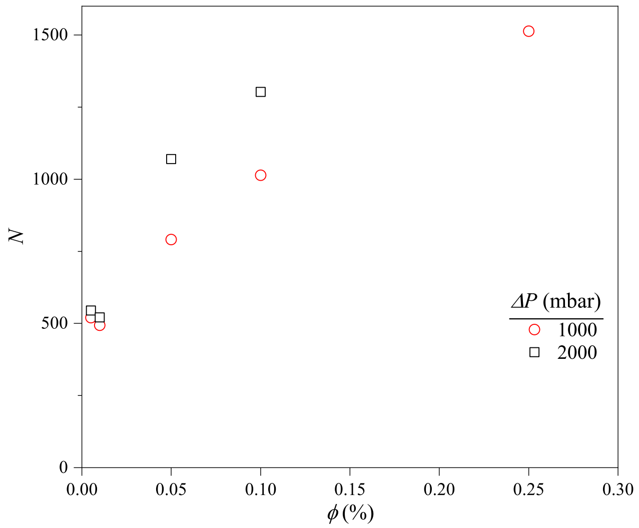

Figure S4. Number of clogging events vary linearly as ϕ for flow tests conducted at ΔP.

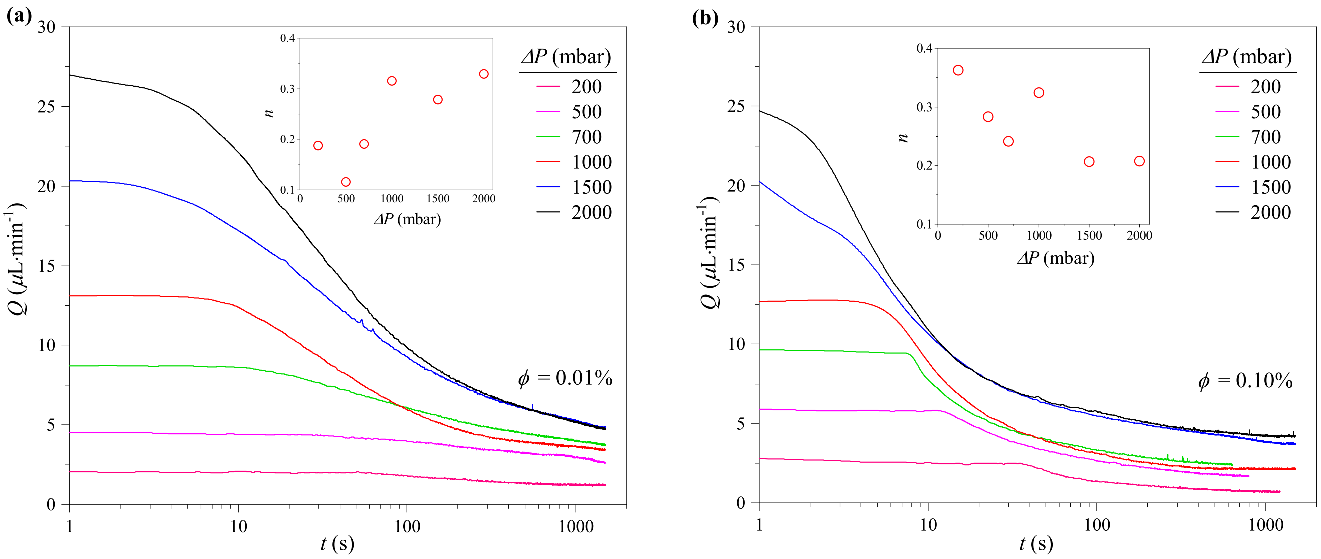

Figure S5. Suspension flowrate decays at different ΔP for: **(a)** ϕ = 0.01%, and **(b)** 0.10%. Inset: Exponents, n, of the power-law decays ($Q \backsim t^{-n}$) plotted as a function of ΔP. The value of n slightly increased with increasing ΔP when ϕ = 0.01%, and decreased when ϕ = 0.10%.

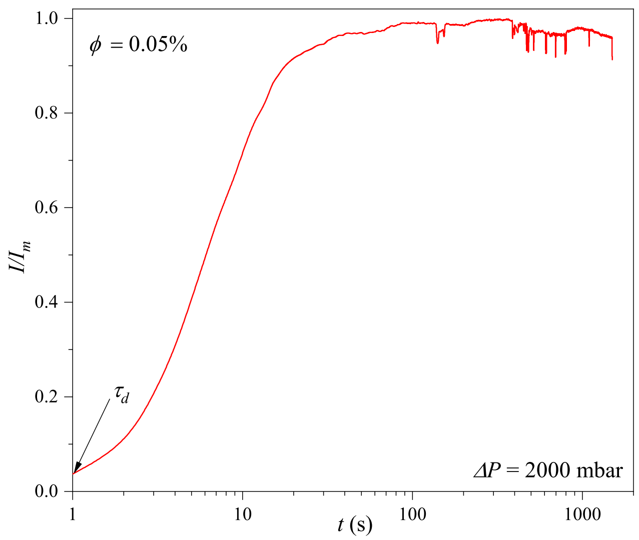

Figure S6. Evolution of fluorescence intensity (I/I_m) at the midpoint of the channels exit, when ΔP = 2000 mbar and ϕ = 0.05%.

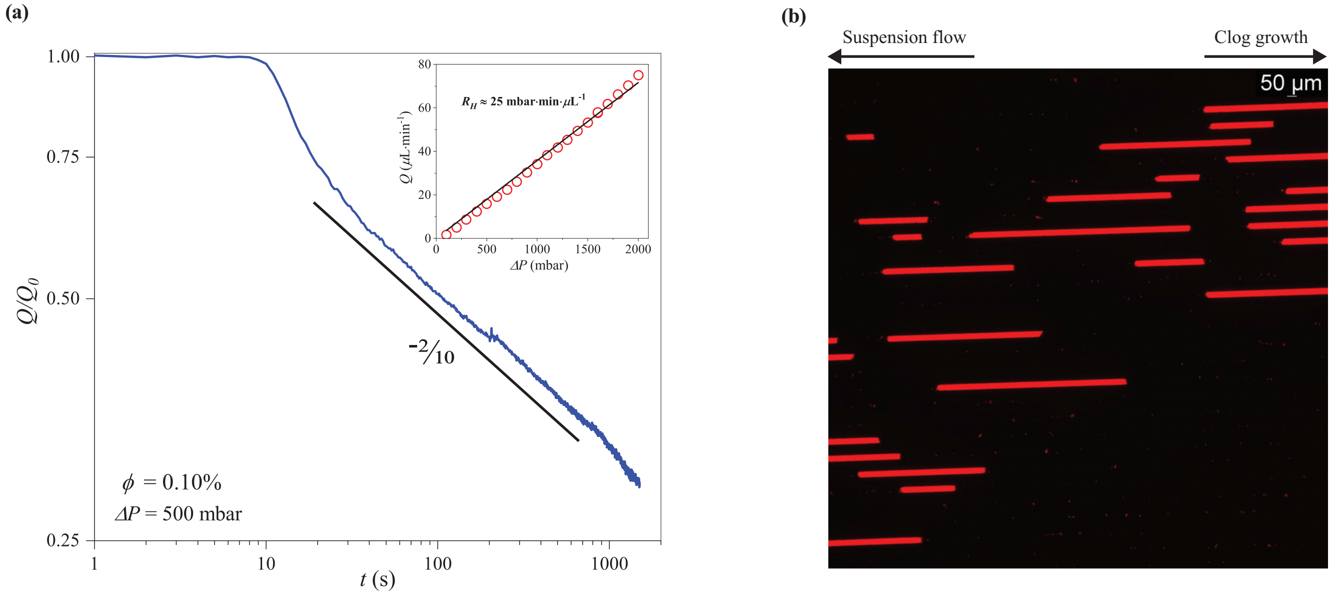

Figure S7. Flow dynamics and clogging in 22.5 μm deep microchannels for ϕ = 0.10% and ΔP = 500 mbar: **(a)** Normalized flowrate decay. Initial flowrate, $Q_0 \approx 16\ \mu$L·min^{-1} and power-law exponent, $n \approx 0.2$. Inset: Estimation of hydraulic resistance ($R_H \approx 25$ mbar·min·μL^{-1}). **(b)** Micrograph showing some clogged channels with multiple distinct clogs.

Table S1. Hydraulic resistances, timescales and exponents of the power-law decays, and post-clogging Darcy permeability of the microfluidic devices.

					n[a]	
	ϕ (%)	R_H (mbar·min·μL^{-1})	τ_d (s)	τ_f (s)	Rapid decay[b]	Slow decay[c]
	0.005	78.13	11	400 – 600	0.28	0.16
	0.01	76.92	10	400 – 600	0.32	0.13
ΔP = 1000 mbar	0.05	80.00	6	500	0.33	-
	0.10	78.13	5	500	0.32	-
	0.25	70.42	4	500	0.27	-

					n		
	ΔP (mbar)	R_H (mbar·min·μL^{-1})	τ_d (s)	τ_f (s)	Rapid decay	Slow decay	κ (μm^2)
	200	89.29	60	400 – 600	0.19	0.15	
	500	107.53	17	400 – 600	0.12	0.14	0.60
	700	78.74	13	400 – 600	0.19	0.17	
ϕ = 0.01%	1000	76.92	10	400 – 600	0.32	0.13	
	1500	78.74	2	400 – 600	0.28	0.20	0.32
	2000	81.97	1	400 – 600	0.33	0.23	

					n		
	ΔP (mbar)	R_H (mbar·min·μL^{-1})	τ_d (s)	τ_f (s)	Rapid decay	Slow decay	κ (μm^2)
	200	84.03	52	500	0.37	-	
	500	82.64	17	500	0.25	-	0.46
	700	90.09	13	500	0.27	-	
ϕ = 0.05%	1000	80.00	6	500	0.33	-	
	1500	81.30	2	400 – 600	0.26	0.14	0.27
	2000	78.74	1	400 – 600	0.23	0.15	

					n		
	ΔP (mbar)	R_H (mbar·min·μL^{-1})	τ_d (s)	τ_f (s)	Rapid decay	Slow decay	κ (μm^2)
	200	72.46	40	600	0.36	-	
	500	81.30	12	600	0.28	-	0.40
	700	71.43	8	600	0.24	-	
ϕ = 0.10%	1000	78.13	5	500	0.32	-	
	1500	78.74	2	600	0.21	-	0.26
	2000	79.37	1	600	0.21	-	

[a] The power-law exponent in $Q \backsim t^{-n}$.

[b] The power-law exponent, n, corresponding to the clog growth timescale.

[c] The power-law exponent, n, associated with the point of inflection on the decay curve when a final plateau was not reached. If a plateau is reached beyond τ_f, '-' is shown in lieu of a value for n.
